# α-linolenic acid and docosahexaenoic acid, alone and combined with trastuzumab, reduce HER2-overexpressing breast cancer cell growth but differentially regulate HER2 signaling pathways

**DOI:** 10.1186/s12944-015-0090-6

**Published:** 2015-08-18

**Authors:** Julie K. Mason, Sukhpreet Klaire, Shikhil Kharotia, Ashleigh K A Wiggins, Lilian U. Thompson

**Affiliations:** Department of Nutritional Sciences, Faculty of Medicine, University of Toronto, 150 College Street, Toronto, ON M5S 3E2 Canada

**Keywords:** Breast cancer, Drug-diet interaction, HER2, Trastuzumab, α-linolenic acid, Docosahexaenoic acid

## Abstract

**Background:**

Diets rich in the n-3 fatty acid alpha-linolenic acid (ALA) have been shown to reduce breast tumor growth, enhance the effectiveness of the HER2-targeted drug trastuzumab (TRAS) and reduce HER2 signaling in mouse models. It is unclear whether this is due to direct effects of ALA or due to its long-chain n-3 fatty acids metabolites including docosahexaenoic acid (DHA).

**Methods:**

The ability of HER2-overexpressing BT-474 human breast cancer cells to convert ALA to long-chain n-3 fatty acids was determined by measurement of phospholipid fatty acids by gas chromatography following treatment with 100 μM ALA. The effects of 96 h treatment with ALA or DHA, at serum levels seen in mice (50–100 μM), alone and combined with TRAS (10 μg/ml), on BT-474 cell growth measured by trypan blue exclusion, apoptosis measured by flow cytometric analysis of Annexin-V/7-AAD stained cells (ALA and TRAS treatment only) and protein biomarkers HER2 signaling measured by western blot were determined.

**Results:**

ALA-treated BT-474 cells had higher phospholipid ALA but no increase in downstream n-3 metabolites including DHA. Both ALA and DHA reduced cell growth with and without TRAS. ALA had no effect on apoptosis. ALA and DHA showed opposite effects on Akt and MAPK phosphorylation; ALA increased and DHA decreased phosphorylation.

**Conclusions:**

Together these data suggest that, while both ALA and its DHA metabolite can reduce HER2-overexpressing breast cancer growth with and without TRAS, they demonstrate for the first time that DHA is responsible for the effects of ALA-rich diets on HER2 signaling pathways.

## Background

Dietary approaches to improve breast cancer outcomes are of interest to the scientific, medical and patient communities [1–3]. n-3 polyunsaturated fatty acids (PUFAs) have anticancer effects in experimental models and are commonly consumed by breast cancer patients [3–5]. α-linolenic acid (ALA, 18:3n-3), found in plant sources such as nuts and seeds, particularly flaxseed (FS), cannot be synthesized in the body. ALA can be metabolized to the long chain n-3 PUFAs found in fish including eicosapentaenoic acid (EPA; 20:5n-3) and docosahexaenoic acid (DHA: 22:6n-3). Whether ALA has independent effects in breast cancer or whether its benefits are due to its long-chain n-3 PUFAs metabolites remains unclear [6]. This is important to establish because it will lead to better dietary recommendations for the use of n-3 PUFAs as complementary treatments in breast cancer.

Animal studies using ALA-rich FS and FS oil (FSO) diets suggest that plant-based n-3 PUFA sources reduce breast cancer growth [7–10]. This effect is subtype-dependent as FS and FSO alone do not affect the growth of HER2-overexpressing BT-474 tumors in athymic mice [11, 12]. Interestingly, FSO diets at 4 % [11] and 8 % [13] levels enhance the effectiveness of the HER2-targeted drug trastuzumab (TRAS) in reducing tumor growth and cell proliferation and increasing apoptosis of BT-474 tumors. This beneficial interaction is at least partially due to reduced signaling through HER2 as indicated by reduced activation of HER2, Akt and MAPK [11]. FSO has also been shown to reduce MAPK and Akt signaling in MCF-7 xenografts [7, 9]. Together, this suggests that ALA-rich FSO may be a beneficial complementary treatment approach in breast cancer.

Serum and tumor levels of ALA and long chain n-3 PUFAs EPA and DHA are significantly higher in mice fed 4 % FSO or 10 % FS diets compared to corn oil-based basal diets [7, 11, 12, 14]. DHA and DHA-rich fish oil have demonstrated anticancer effects [15–20] and have also been shown to affect HER2 signaling [17] in models of HER2 overexpressing breast cancer. It is therefore of interest to determine the independent effects of ALA and DHA at serum levels seen after FSO feeding and to explore their interaction with TRAS.

It is suggested that breast cancer cell lines lack the ability to convert ALA to its downstream metabolites, importantly EPA and DHA due to a lack of Δ6 desaturase enzyme. This has been demonstrated in MCF-7, MDA-MB-231 and T47D cells where downstream products of reactions catalyzed by of Δ6 desaturase including gamma linolenic acid, stearidonic acid and DHA were not produced following treatment with their precursors linoleic acid, ALA, and EPA, respectively [21–23]. In the current study we have found for the first time that BT-474 cells are also unable to metabolize ALA to DHA, thus presenting a good model to study the independent effects of ALA and DHA.

Our overall objective was to determine using cell culture methods whether ALA is the component of FSO responsible for the effects seen in vivo on HER2 signaling in BT-474 xenografts. Specifically, we aimed to determine the effect of ALA with and without TRAS on cell growth, phospholipid fatty acid profile and protein biomarkers of HER2 signaling in BT-474 cells. Subsequently, the effects of physiologically relevant doses of the ALA metabolite, DHA, alone and combined with TRAS, on cell growth and HER2 signaling biomarker expression were measured. Overall, our findings demonstrate that both ALA and its DHA metabolite reduce BT-474 cell growth, however, show for the first time that only DHA reduces activation of proteins in HER2 signaling pathways. This finding is significant because it emphasizes the differences in mechanisms whereby n-3 PUFAs exert their effect in breast cancer.

## Methods

### Cell Line

The BT-474 (HTB-20) cell line was obtained from the American Type Cell Culture (ATCC; Manassas, VA, USA) which adheres to the highest ethical standards for obtaining human tissues. Cells were authenticated by STR analysis at the Centre for Applied Genomics at the Sickkids Research Institute (Toronto, ON). Cells were maintained in RPMI medium (Gibco, Carlsbad, CA, USA) supplemented with 10 % fetal bovine serum (FBS; Sigma-Aldrich, St. Louis, MO, USA) and 1 % antibiotic-antimycotic solution containing penicillin, streptomycin and amphotericin B (Gibco).

### Fatty acid and TRAS treatment

ALA and DHA (>99 % pure) were obtained from Sigma-Aldrich. Stock solutions were prepared in 100 % ethanol, aliquoted, flushed with nitrogen gas and stored at −20 °C. In preparation for treatment, ethanol was evaporated with nitrogen gas. 4 mM solutions were prepared in charcoal-stripped FBS (CS-FBS; Sigma-Aldrich) and incubated for 1 h at 37 °C to allow for the fatty acids to complex with albumin in the CS-FBS. TRAS was purchased from the Princess Margaret Cancer Centre Pharmacy (Toronto, ON). A 20 mg/ml stock solution of TRAS was prepared every two weeks using bacteriostatic water and stored at 4 °C.

Cells in the log phase of growth were plated in tissue culture plates or flasks (Sarstedt, Numbrecht, Germany) in RPMI maintenance medium. After approximately 72 h, the medium was removed and replaced with treatment medium. Treatment solutions were prepared using phenol red-free RPMI 1640 medium (Gibco) containing 1 % antibiotics-antimycotics, 5 % CS-FBS and 1 nM 17β-estradiol (E2; Sigma-Aldrich) dissolved in ethanol. The control treatment medium contained no fatty acids or TRAS. TRAS treatment was at 10 μg/ml, a dose commonly used in vitro [24, 25] and a target serum trough concentration in clinical studies [26]. In experiment 1, cells were treated with ALA (50 and/or 100 μM) alone and combined with TRAS and cell growth, apoptosis, phospholipid fatty acids and HER2 signaling protein biomarkers were measured. In experiment 2, cells were treated with DHA (50 and 100 μM) alone and combined with TRAS and cell growth and HER2 signaling protein biomarkers were measured.

In both experiments, treatment medium was refreshed after 48 h. To demonstrate that the effect was not simply due to the addition of fatty acids to the medium, an additional experiment was conducted to confirm the effect of ALA on cell growth using a background of other fatty acids (40 μM oleic acid and 40 μM linoleic acid) in both the control and treatment groups.

### Cell growth

After a total treatment period of 96 h, cells grown in 24 well plates were harvested in 0.25 % trypsin-EDTA (Sigma-Aldrich). Cell number was counted using the TC20 automated cell counter (Bio-Rad, Hercules, CA, USA) based on the trypan blue exclusion assay. The total live cell number for each well was recorded and the average of the 3 technical replicates was taken. The experiments were repeated 3–5 times. Results are presented as a percentage of the untreated control cell number.

### Apoptosis

After 96 h treatment, cells grown in 6 well plates (Sigma) were collected and washed in phosphate buffered saline (PBS). Cells were then incubated with 5 μL Annexin V-APC and 7-AAD stain (BD Biosciences, Mississauga, ON) for 15 min at room temperature in the dark. Controls were prepared by adding no stains, Annexin V only and 7-AAD only. 400 μl of binding buffer (BD Biosciences) was added to the samples. Samples were immediately analyzed by flow cytometry using a BD LSR Fortessa. Viable (Annexin V-/7-AAD-) and apoptotic cells (Annexin V+/7-AAD- and Annexin V+/7-AAD+) were quantified as a percent of total cells.

### Protein signaling biomarkers

Cells were treated for 48 and/or 96 h and then protein was extracted using RIPA lysis buffer (Cell Signaling Technology, Beverley, MA, USA) containing protease inhibitors (Complete Mini, EDTA-free Protease Inhibitor Cocktail Tablets, Roche) and phosphatase inhibitors (PhosSTOP Phosphatase Inhibitor Cocktail Tablets, Roche). Protein concentration was measured using the bicinchoninic acid assay (Thermo Fisher, Ottawa, ON). Proteins solubilized in Laemmli buffer containing β-mercaptoethanol (Bio-Rad) were heated for 5 min at 95 °C and separated in 7.5-10 % polyacrylamide gels (TGX, Bio-Rad) and transferred to polyvinylidene difluoride membranes. Membranes were blocked with blocking buffer (5 % BSA or 5 % non-fat dry milk in Tris-buffered saline/0.1 % Tween-20 (TBS-T), Cell Signaling Technology) for 1 h, washed in TBS-T and then incubated with primary antibody overnight in a 5 % BSA solution. Primary antibodies included HER2 (CST 2165), pHER2 (CST 4290), Akt1 (CST 2938), pAkt1 (CST 9018), MAPK (MAPK; CST 4695), pMAPK (CST 4377), caspase-3 (CST 9665) and β-actin (CST 3700). After 3 washes in TBS-T, membranes were incubated with anti-rabbit horseradish peroxidase-conjugated secondary antibody and developed with LumiGLO® chemiluminescent reagent and peroxide (Cell Signaling Technology). Signal was detected on X-ray film (Clonex Corporation, Markham, ON) using a Konica Minolta™ SRX-101A Film Processor. Band intensity was quantified using ImageJ Software (National Institutes of Health, Bethesda). The “relative expression” of each biomarker was calculated by dividing each sample’s biomarker band intensity by its β-actin band intensity (from stripped and re-probed membrane). The ratios of phosphorylated to total HER2, Akt1 and MAPK were calculated as an indicator of HER2 signaling pathway activation.

### Phospholipid fatty acids

After 96 h of treatment, cells were trypsinized, washed twice in PBS and resuspended in fatty acid free medium. Total lipids were extracted as described by Bligh and Dyer [27] using NaCl, methanol and choloform (Sigma-Alrich) in a 1:2:2 ratio. Thin-layer chromatography was used to separate lipid classes using silica G-plates (EMD Chemical, Gibbstown, NJ, USA) Total phospholipid fatty acids were collected into test tubes and a known amount of 17:0 standard (Avanti, Alabaster, AL, USA) was added. Fatty acids were converted to fatty acid methyl esters (FAME) by incubating for 1 h at 100 °C in hexane and boron trifluoride-methanol (Sigma-Aldrich). FAME were transferred to gas chromatography (GC) vials and samples were analyzed by GC-flame ionization detection (GC-FID) using a Varian-430 GC (Varian, Lake Forest, CA, USA) equipped with a Varian FactorFour capillary column (VF-23 ms; 30 m · 0.25 mm i.d. · 0.25 lm film thickness) and a FID. Samples were injected in splitless mode as previously described [28]. Fatty acids were identified by comparison to a reference standard consisting of GLC-68 and GLC-455 supplemented with 8:0, 10:0, and 12:0 methyl esters (Nu-Chek Prep, Elysian, MN) and quantified by comparing the area of the peaks to the 17:0 peak. Results are presented as mole percentages of total fatty acids.

### Statistical analysis

Statistical analyses were performed using SigmaPlot (version 12.0; Systat Software Inc., San Jose, CA). Data are expressed as mean ± SEM. Unpaired *t*-test was used to determine differences in phospholipid fatty acids between control and ALA treated cells. For all other outcomes, two way analysis of variance was used to evaluate main effects of fatty acids (ALA or DHA), TRAS and their interactions. If significant interactions were observed (P < 0.1), Tukey’s post-hoc test was used to compare each treatment group with significance set at P < 0.05.

## Results

### Experiment 1. Effect of ALA with and without TRAS

#### Cell growth and apoptosis

**Fig. 1:**
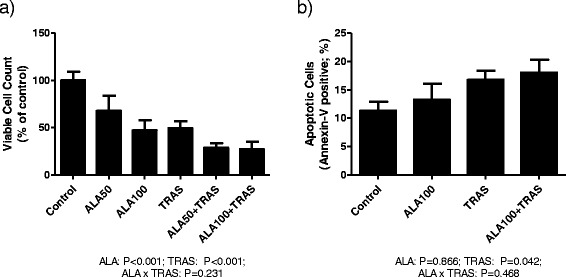
Effect of ALA and TRAS, alone and in combination, on the (**a**) growth and (**b**) apoptosis of BT-474 cells. n = 3-5

TRAS had main effects in reducing cell growth (Fig. 1a) and increasing the number of apoptotic (annexin-V positive) cells (Fig. 1b) while ALA had main effect in reducing cell growth (Fig. 1a). The same growth reduction results as those shown in Fig. 1 were observed when ALA was tested on a background of other fatty acids (data not shown) indicating that it is not simply the presence of fatty acids that is affecting cell growth. ALA did not significantly affect the number of apoptotic cells based on annexin-V staining (Fig. 1b). Since there was a small but non-significant increase in apoptotic cells in the ALA treated cells compared to control, caspase 3 protein expression was measured by western blot but no difference in total or cleaved protein expression was detected (data not shown).

#### Protein biomarkers of HER2 signaling pathways

**Fig. 2:**
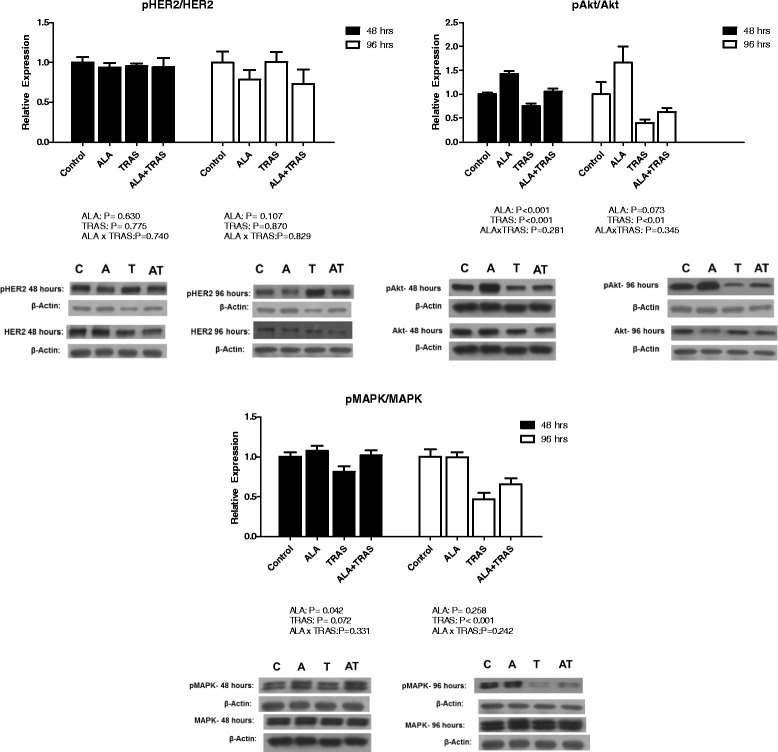
Effect of ALA and TRAS, alone and in combination, on the expression of total and phosphorylated HER2, Akt and MAPK after 48 and 96 h of treatment of BT-474 cells. (n = 5-6). Representative blots are displayed

Figure 2 shows that pHER2/HER2 expression was not affected by ALA and TRAS alone and in combination at 48 and 96 h. TRAS significantly reduced pAkt/Akt (48 and 96 h) and pMAPK/MAPK (96 h). ALA treatment resulted in significantly higher pAkt/Akt and pMAPK/MAPK at 48 h but had no significant effect at 96 h. ALAxTRAS interactions were not seen for any biomarkers at any time point.

#### Phospholipid fatty acid profile

TRAS had no effect on the fatty acid composition of the cells. Therefore, the results were pooled for cells treated with 0 uM ALA (control and TRAS) and with 100 μM ALA (ALA and ALA + TRAS). As seen in Table 1, ALA treatment caused a dramatic increase in phospholipid ALA (18:3n-3; 4926 %) but no formation of ALA metabolites; in fact, levels of EPA and DHA were significantly lower in ALA-treated cells.

**Table 1:** Phospholipid fatty acid profile of control and ALA-treated BT-474 cells. Results are presented as molar % of total fatty acids. *N* = 6

	Control	ALA
14:0	2.03 ± 0.62	1.27 ± 1.08
16:0	28.95 ± 4.94	27.16 ± 4.68
18:0	18.11 ± 3.14	17.95 ± 2.62
20:0	0.94 ± 0.21	0.56 ± 0.13
22:0	0.10 ± 0.03^a^	1.14 ± 0.10^b^
16:1 n-7	6.97 ± 2.86	6.24 ± 3.55
18:1n-9	16.45 ± 1.03	11.74 ± 1.62
18:1n-7	4.36 ± 0.18^a^	2.68 ± 0.34^b^
20:1n-9	0.32 ± 0.08	0.05 ± 0.02
22:1n-9	1.35 ± 0.70^a^	0.53 ± 0.20^b^
18:2n-6	4.68 ± 0.86	3.11 ± 0.42
18:3n-6	1.95 ± 0.92	1.15 ± 0.29
20:2n-6	0.35 ± 0.05^a^	0.18 ± 0.03^b^
20:4n-6	6.34 ± 0.63^a^	2.83 ± 0.79^b^
22:4n-6	0.38 ± 0.05^a^	0.14 ± 0.02^b^
22:5n-6	0.08 ± 0.04	0.04 ± 0.01
18:3n-3	0.39 ± 0.15^a^	19.60 ± 3.60^b^
20:3n-3	2.64 ± 0.22^a^	1.09 ± 0.10^b^
20:5n-3	1.29 ± 0.81	1.14 ± 0.19
22:5n-3	1.18 ± 0.06	0.84 ± 0.17
22:6n-3	1.15 ± 0.12^a^	0.55 ± 0.14^b^
n-6:n-3	2.09 ± 0.08^a^	0.34 ± 0.07^b^

*values with different superscripts in the same row are significantly different by unpaired *T*-test

### Experiment 2. Effect of DHA with and without TRAS

#### Cell growth

**Fig. 3:**
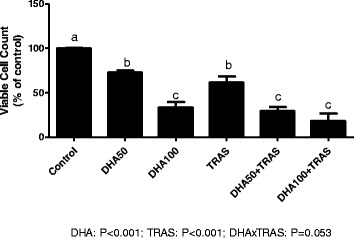
Effect of DHA and TRAS, alone and in combination, on the growth of BT-474 cells. n = 3

Both DHA and TRAS showed main effects in reducing the growth of BT-474 cells (Fig. 3). There was a significant DHAxTRAS interaction. Multiple comparisons showed a significant dose-dependent reduction with DHA alone. 50 and 100 μM DHA combined with TRAS significantly reduced cell growth compared to TRAS alone and there was no significant difference between the two DHA doses.

#### Protein biomarkers of HER2 signaling pathways

**Fig. 4:**
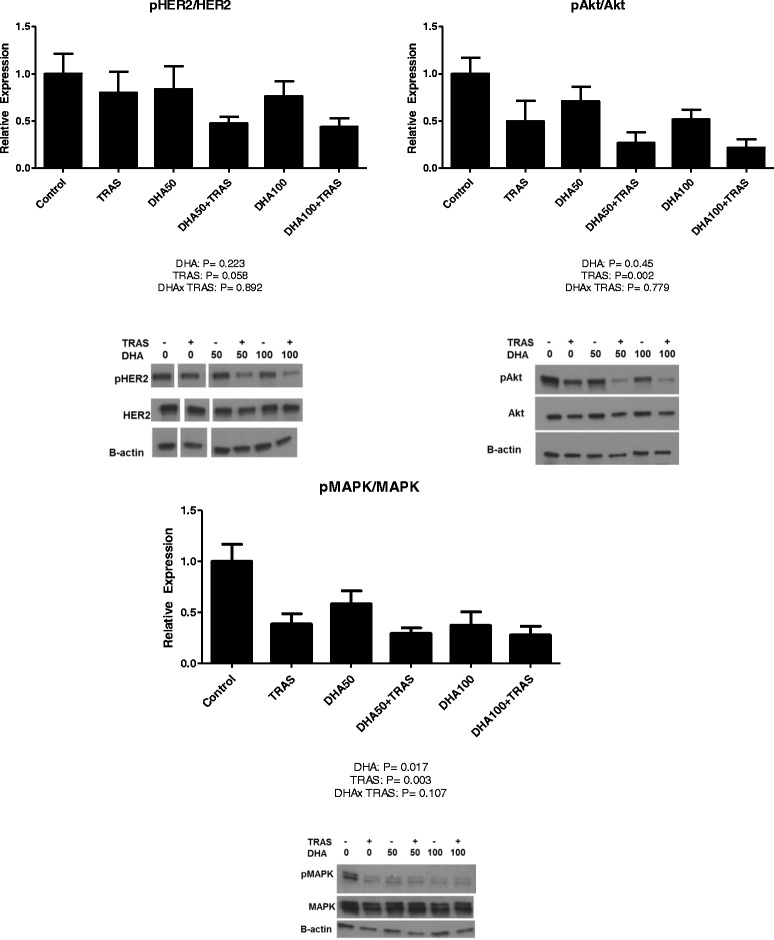
Effect of DHA (50 and 100 μM) and TRAS, alone and in combination, on the expression of total and phosphorylated HER2, Akt and MAPK after 96 h of treatment of BT-474 cells. (n = 5-6). Representative blots are displayed. For HER2 protein biomarkers, blot images were cropped and rearranged for consistency of presentation of treatment groups. Bands for each biomarkers are from the same blot

Neither DHA nor TRAS had main effects on pHER2/HER2 expression nor did they show any interaction (Fig. 4). Although not statistically significant, there was 45 % lower pHER2/HER2 expression in the DHA100 + TRAS treated cells compared to TRAS treated cells. There were significant main effects of TRAS in reducing the expression of pMAPK/MAPK and pAkt/Akt. DHA had main effects in reducing pMAPK/MAPK and pAkt/Akt. DHA + TRAS100 resulted in 27 % lower pMAPK/MAPK and 56 % lower pAkt/Akt compared to TRAS alone.

## Discussion

This study has shown that both ALA and DHA reduce the growth of BT-474 cells and enhance the effect of TRAS in reducing cell growth. Our first experiment showed that BT-474 cells lack the ability to convert ALA to downstream metabolites thereby presenting a good system to study the independent effects of ALA and its metabolite DHA. The results strongly suggest that DHA reduces growth factor receptor signaling as indicated by reductions in phosphorylation of Akt and MAPK while the opposite effect is seen for the plant-based n-3 PUFA ALA. In vitro ALA treatment reduced phospholipid DHA level and increased phosphorylation of Akt and MAPK, thus it is possible that the in vitro effect of ALA on Akt and MAPK activation is driven by this decrease in phospholipid DHA. It is important to note that feeding an ALA-rich diet does not decrease but rather increases tumor DHA level in mice [11, 12] suggesting that this loss of DHA is isolated to in vitro models.

The mechanism driving the growth reducing effect of ALA in this HER2 overexpressing cell line requires further elucidation. Based on the current study, ALA is not acting through an induction of apoptosis or reduction of HER2 signaling pathways. Although the effect of ALA on breast cancer cell growth has been previously explored [7, 21, 29–32], only two studies have looked at effects on HER2-overexpressing BT-474 cells [33, 34]. Similar to our findings, ALA alone [34, 33] and combined with TRAS (5 μg/ml) [33] reduced BT-474 cell growth; however, Menendez et al. saw significant reductions in HER2 mRNA and protein following treatment with 20 μM ALA in that study. Several, but not all, studies looking at the effect of ALA in other breast cancer cell lines have shown significant growth reductions [7, 21, 29–32]. Though apoptosis has been suggested as a mechanism of ALA effect in MCF-7 cells [29], no such effect of ALA on apoptosis was seen in BT-474 cells. It is possible that ALA modulates cell growth through estrogen receptor (ER)-related mechanisms. ALA has been shown to bind to the α and β isoforms of the estrogen receptor and exert mixed agonistic/agonistic effects at different doses [32]. Dietary FSO reduces the expression of E2-sensitive genes including progesterone receptor (PGR) and cyclin D1 in MCF-7 xenografts [9, 35], an effect that we also have shown with ALA treatment in vitro in MCF-7 cells (unpublished work). Further research is needed to determine the mechanism for ALA effect in breast cancer.

More studies have investigated the effect of DHA on cell growth and apoptosis [16, 18, 22, 31, 36–41]. Others have similarly found that DHA reduces the growth of HER2 overexpressing breast cancer cells (BT-474 and SkBr-3) [17, 18]. Ewaschuk et al. [18] studied the effect of DHA with and without TRAS on SkBr-3 cells. They found a 27 % reduction with TRAS treatment alone yet when 100 μM DHA was combined with TRAS a much greater reduction was observed (63 %). Similar to our findings, there was no significant difference between the cells treated with 100 μM DHA in the absence or presence of TRAS [18]. Others have suggested that DHA affects cell signaling pathways through incorporation into membrane rafts thereby affecting the distribution and activity of associated receptors [22, 36, 38]. DHA has been fairly consistently shown to induce apoptosis or affect the expression of markers involved in apoptosis pathways in breast cancer cells [22, 39, 42–45]. Furthermore, it is well established that treatment with DHA increases phospholipid DHA increases in breast cancer cells [21, 22, 42]. Apoptosis and fatty acid analysis following DHA treatment was therefore not conducted in the current study.

We acknowledge several limitations of the current study. One limitation is that only one ALA metabolite was studied. EPA is another n-3 ALA metabolite but we focused specifically on DHA for a number of reasons. EPA is a minor constituent of tumor and serum fatty acids [7, 11, 12, 14]. Feeding of 10 % FS or 4 % FSO resulted in serum levels of only 15–22 μM EPA [7, 14] while studies looking at EPA effect on cell growth in vitro have seen growth reduction at or above approximately 100 μM [18, 22, 37]. On the other hand, DHA concentration in the serum of 10 % FS and 4 % FSO-fed mice was shown to be 244 and 193 μM [7, 14], respectively, while DHA has been shown to reduce breast cancer growth at doses as low as 25 μM in HER2-overexpressing SkBr-3 cells [18]. Furthermore, there was support for the hypothesis that DHA affects HER2 signaling pathways [16, 17]. An additional limitation is that only one cell line was used in the current study. However, literature suggests that the DHA and ALA effects observed in our study are likely not cell-line specific since DHA alone has been shown to reduce pAkt in both BT-474 and SkBr-3 cells [17] and DHA has been shown to enhance TRAS effect in SkBr-3 cells [18]. Furthermore, work from our research group has shown that ALA treatment reduces the growth of four breast cancer cell lines with varying receptor expression [34].

This study was conducted to clarify whether ALA or its metabolite, DHA, caused the effects seen in vivo where a diet with ALA-rich FSO significantly enhanced the effectiveness of TRAS [11]. Therefore, n-3 fatty acid concentrations used were derived from studies in the athymic mouse model with feeding of 4 % FS oil or 10 % FS diets. Serum ALA levels of mice fed these diets ranged from 33 to 108 μM [7, 14]. Hence, in the current study, cells were treated with 50 and 100 μM ALA, levels within this range. In mice fed a 10 % FS or 4 % FSO diet, approximately 100–150 μM higher serum DHA was detected compared to mice fed the basal diet*.* However we did not treat the cells with 150 uM DHA as preliminary results from our lab suggested that DHA treatment higher than 100 μM was cytotoxic. Thus we treated the cells with 50 and 100 μM. Overall, our findings suggest that treating BT-474 cells with serum levels of ALA seen in our animal model reduces cell growth with and without TRAS but does not match the effects on HER2 signaling pathway markers seen in vivo. On the other hand, treating BT-474 cells with the concentration of DHA seen following FSO feeding reduces cell growth and biomarkers of the HER2 signaling pathway in a similar manner to our in vivo study.

Humans are known to be poor converters of ALA to DHA and it is suggested that the best way to increase serum levels of DHA is through dietary intake [46]. Several factors are suggested to affect this conversion including background fatty acids in the diet and sex [46, 47]. Daily consumption of approximately 6 g of ALA from FSO for 12 weeks has been shown to increase serum ALA by approximately 154 μM but increase DHA only by 15 μM [48]. Our findings suggest that interventions that significantly increase serum DHA are required for modulation of HER2 signaling pathway. Interestingly, a randomized controlled trial showed that consumption of 25 g of FS per day, providing approximately 6 g of ALA, by breast cancer patients significantly reduced cell proliferation and HER2 expression [49]. This suggests that despite the low conversion to DHA in humans, ALA-rich diets may significantly reduce breast tumor growth in breast cancer patients.

The overall objective of this study was to determine whether ALA is the component of FSO responsible for the effects seen in vivo on HER2 signaling in BT-474 xenografts. ALA alone did not cause significant downregulation of HER2 signaling while DHA did; therefore, our findings suggest that the effects of FS and FSO seen in animal studies on growth factor signaling pathways is likely due to DHA produced from hepatic conversion of ALA to DHA and not due to ALA itself. Despite this lack of effect on growth factor receptor signaling, ALA significantly reduced cell growth perhaps by different mechanisms including through estrogen receptor signaling, which merits further exploration. Our findings suggest that there are differences in the mechanisms of ALA and DHA growth effects in HER2 overexpressing cells. These significant findings contribute to our understanding of the role of n-3 PUFAs in breast cancer and may help in the development of nutritional approaches for breast cancer treatment.

## Abbreviations

ALA: α-linolenic acid
CS-FBS: Charcoal-stripped fetal bovine serum
DHA: Docosahexaenoic acid
E2: 17β-estradiol
EPA: Eicosapentaenoic acid
ER: Estrogen receptor
FAME: Fatty acid methyl ester
FS: Flaxseed
FSO: Flaxseed oil
HER2: Human epidermal growth factor receptor 2
PUFA: Polyunsaturated fatty acid
TRAS: Trastuzumab

## References

[1] World Cancer Research Fund/American Institute for Cancer Research. Food, Nutrition, Physical Activity, and the Prevention of Cancer: a Global Perspective. Washington DC: 2007.

[2] Eccles SA, Aboagye EO, Ali S, Anderson AS, Armes J, Berditchevski F (2013). Critical research gaps and translational priorities for the successful prevention and treatment of breast cancer. Breast Cancer Res.

[3] Boon HS, Olatunde F, Zick SM (2007). Trends in complementary/alternative medicine use by breast cancer survivors: comparing survey data from 1998 and 2005. BMC Womens Health.

[4] Boucher BA, Cotterchio M, Curca IA, Kreiger N, Harris SA, Kirsh VA (2012). Intake of phytoestrogen foods and supplements among women recently diagnosed with breast cancer in Ontario. Canada Nutr Cancer.

[5] Brasky TM, Lampe JW, Potter JD, Patterson RE, White E (2010). Specialty supplements and breast cancer risk in the VITamins And Lifestyle (VITAL) Cohort. Cancer Epidemiol Biomarkers Prev.

[6] Anderson BM, Ma DW (2009). Are all n-3 polyunsaturated fatty acids created equal?. Lipids Health Dis.

[7] Truan JS, Chen JM, Thompson LU (2010). Flaxseed oil reduces the growth of human breast tumors (MCF-7) at high levels of circulating estrogen. Mol Nutr Food Res.

[8] Wang L, Chen J, Thompson LU (2005). The inhibitory effect of flaxseed on the growth and metastasis of estrogen receptor negative human breast cancer xenograftsis attributed to both its lignan and oil components. Int J Cancer.

[9] Saggar JK, Chen J, Corey P, Thompson LU (2010). The effect of secoisolariciresinol diglucoside and flaxseed oil, alone and in combination, on MCF-7 tumor growth and signaling pathways. Nutr Cancer.

[10] Chen J, Saggar JK, Corey P, Thompson LU (2009). Flaxseed and pure secoisolariciresinol diglucoside, but not flaxseed hull, reduce human breast tumor growth (MCF-7) in athymic mice. J Nutr.

[11] Mason JK, Fu M, Chen J, Thompson LU (2015). Flaxseed oil enhances the effectiveness of trastuzumab in reducing the growth of HER2-overexpressing human breast tumors (BT-474). J Nutr Biochem.

[12] Mason JK, Fu MH, Chen J, Yu Z, Thompson LU (2013). Dietary flaxseed-trastuzumab interactive effects on the growth of HER2-overexpressing human breast tumors (BT-474). Nutr Cancer.

[13] Mason JK, Chen J, Thompson LU (2010). Flaxseed oil-trastuzumab interaction in breast cancer. Food Chem Toxicol.

[14] Mason JK, Kharotia S, Wiggins AK, Kitson AP, Chen J, Bazinet RP (2014). 17beta-estradiol increases liver and serum docosahexaenoic acid in mice fed varying levels of alpha-linolenic acid. Lipids.

[15] Xue M, Wang Q, Zhao J, Dong L, Ge Y, Hou L (2014). Docosahexaenoic acid inhibited the Wnt/beta-catenin pathway and suppressed breast cancer cells in vitro and in vivo. J Nutr Biochem.

[16] Cao W, Ma Z, Rasenick MM, Yeh S, Yu J (2012). N-3 poly-unsaturated fatty acids shift estrogen signaling to inhibit human breast cancer cell growth. PLoS One.

[17] Zou Z, Bellenger S, Massey KA, Nicolaou A, Geissler A, Bidu C (2013). Inhibition of the HER2 pathway by n-3 polyunsaturated fatty acids prevents breast cancer in fat-1 transgenic mice. J Lipid Res.

[18] Ewaschuk JB, Newell M, Field CJ (2012). Docosahexanoic acid improves chemotherapy efficacy by inducing CD95 translocation to lipid rafts in ER(−) breast cancer cells. Lipids.

[19] Leslie MA, Abdelmagid SA, Perez K, Muller WJ, Ma DW (2014). Mammary tumour development is dose-dependently inhibited by n-3 polyunsaturated fatty acids in the MMTV-neu(ndl)-YD5 transgenic mouse model. Lipids Health Dis.

[20] Yee LD, Young DC, Rosol TJ, Vanbuskirk AM, Clinton SK (2005). Dietary (n-3) polyunsaturated fatty acids inhibit HER-2/neu-induced breast cancer in mice independently of the PPARgamma ligand rosiglitazone. J Nutr.

[21] Grammatikos SI, Subbaiah PV, Victor TA, Miller WM (1994). n-3 and n-6 fatty acid processing and growth effects in neoplastic and non-cancerous human mammary epithelial cell lines. Br J Cancer.

[22] Corsetto PA, Montorfano G, Zava S, Jovenitti IE, Cremona A, Berra B (2011). Effects of n-3 PUFAs on breast cancer cells through their incorporation in plasma membrane. Lipids Health Dis.

[23] Bardon S, Le MT, Alessandri JM (1996). Metabolic conversion and growth effects of n-6 and n-3 polyunsaturated fatty acids in the T47D breast cancer cell line. Cancer Lett.

[24] Wang CX, Koay DC, Edwards A, Lu Z, Mor G, Ocal IT (2005). In vitro and in vivo effects of combination of Trastuzumab (Herceptin) and Tamoxifen in breast cancer. Breast Cancer Res Treat.

[25] Pfeiler G, Horn F, Lattrich C, Klappenberger S, Ortmann O, Treeck O (2007). Apoptotic effects of signal transduction inhibitors on human tumor cells with different PTEN expression. Oncol Rep.

[26] Cobleigh MA, Vogel CL, Tripathy D, Robert NJ, Scholl S, Fehrenbacher L (1999). Multinational study of the efficacy and safety of humanized anti-HER2 monoclonal antibody in women who have HER2-overexpressing metastatic breast cancer that has progressed after chemotherapy for metastatic disease. J Clin Oncol.

[27] Bligh EG, Dyer WJ (1959). A rapid method of total lipid extraction and purification. Can J Biochem Physiol.

[28] Chen CT, Liu Z, Bazinet RP (2011). Rapid de-esterification and loss of eicosapentaenoic acid from rat brain phospholipids: an intracerebroventricular study. J Neurochem.

[29] Kim JY, Park HD, Park E, Chon JW, Park YK (2009). Growth-inhibitory and proapoptotic effects of alpha-linolenic acid on estrogen-positive breast cancer cells. Ann N Y Acad Sci.

[30] Horia E, Watkins BA (2005). Comparison of stearidonic acid and alpha-linolenic acid on PGE2 production and COX-2 protein levels in MDA-MB-231 breast cancer cell cultures. J Nutr Biochem.

[31] Chajes V, Sattler W, Stranzl A, Kostner G (1995). Influence of n-3 fatty acids on the growth of human breast cancer cells in vitro: relationship to peroxides and vitamin-E. Breast Cancer Res Treat.

[32] Tran HN, Bae SY, Song BH, Lee BH, Bae YS, Kim YH (2010). Pomegranate (Punica granatum) seed linolenic acid isomers: concentration-dependent modulation of estrogen receptor activity. Endocr Res.

[33] Menendez JA, Vazquez-Martin A, Ropero S, Colomer R, Lupu R (2006). HER2 (erbB-2)-targeted effects of the omega-3 polyunsaturated fatty acid, alpha-linolenic acid (ALA; 18:3n-3), in breast cancer cells: the “fat features” of the “Mediterranean diet” as an “anti-HER2 cocktail”. Clin Transl Oncol.

[34] Wiggins AKA, Mason JK, Thompson LU. Growth and gene expression differ over time in alpha-linolenic acid treated breast cancer cells. Exp Cell Res. 2015;333(1):147–54. http://dx.doi.org/10.1016/j.yexcr.2015.02.020. 10.1016/j.yexcr.2015.02.020 25743093

[35] Saggar JK, Chen J, Corey P, Thompson LU (2010). Dietary flaxseed lignan or oil combined with tamoxifen treatment affects MCF-7 tumor growth through estrogen receptor- and growth factor-signaling pathways. Mol Nutr Food Res.

[36] Ravacci GR, Brentani MM, Tortelli T, Torrinhas RS, Saldanha T, Torres EA (2013). Lipid raft disruption by docosahexaenoic acid induces apoptosis in transformed human mammary luminal epithelial cells harboring HER-2 overexpression. J Nutr Biochem.

[37] Chamras H, Ardashian A, Heber D, Glaspy JA (2002). Fatty acid modulation of MCF-7 human breast cancer cell proliferation, apoptosis and differentiation. J Nutr Biochem.

[38] Schley PD, Brindley DN, Field CJ (2007). (n-3) PUFA alter raft lipid composition and decrease epidermal growth factor receptor levels in lipid rafts of human breast cancer cells. J Nutr.

[39] Schley PD, Jijon HB, Robinson LE, Field CJ (2005). Mechanisms of omega-3 fatty acid-induced growth inhibition in MDA-MB-231 human breast cancer cells. Breast Cancer Res Treat.

[40] Rogers KR, Kikawa KD, Mouradian M, Hernandez K, McKinnon KM, Ahwah SM (2010). Docosahexaenoic acid alters epidermal growth factor receptor-related signaling by disrupting its lipid raft association. Carcinogenesis.

[41] Ghosh-Choudhury T, Mandal CC, Woodruff K, St Clair P, Fernandes G, Choudhury GG (2009). Fish oil targets PTEN to regulate NFkappaB for downregulation of anti-apoptotic genes in breast tumor growth. Breast Cancer Res Treat.

[42] Blanckaert V, Ulmann L, Mimouni V, Antol J, Brancquart L, Chenais B (2010). Docosahexaenoic acid intake decreases proliferation, increases apoptosis and decreases the invasive potential of the human breast carcinoma cell line MDA-MB-231. Int J Oncol.

[43] Lee EJ, Yun UJ, Koo KH, Sung JY, Shim J, Ye SK (2014). Down-regulation of lipid raft-associated onco-proteins via cholesterol-dependent lipid raft internalization in docosahexaenoic acid-induced apoptosis. Biochim Biophys Acta.

[44] Kang KS, Wang P, Yamabe N, Fukui M, Jay T, Zhu BT (2010). Docosahexaenoic acid induces apoptosis in MCF-7 cells in vitro and in vivo via reactive oxygen species formation and caspase 8 activation. PLoS One.

[45] Xiong A, Yu W, Tiwary R, Sanders BG, Kline K (2012). Distinct roles of different forms of vitamin E in DHA-induced apoptosis in triple-negative breast cancer cells. Mol Nutr Food Res.

[46] Brenna JT, Salem N, Sinclair AJ, Cunnane SC (2009). alpha-Linolenic acid supplementation and conversion to n-3 long-chain polyunsaturated fatty acids in humans. Prostaglandins Leukot Essent Fatty Acids.

[47] Kitson AP, Stroud CK, Stark KD (2010). Elevated production of docosahexaenoic acid in females: potential molecular mechanisms. Lipids.

[48] Austria JA, Richard MN, Chahine MN, Edel AL, Malcolmson LJ, Dupasquier CM (2008). Bioavailability of alpha-linolenic acid in subjects after ingestion of three different forms of flaxseed. J Am Coll Nutr.

[49] Thompson LU, Chen JM, Li T, Strasser-Weippl K, Goss PE (2005). Dietary flaxseed alters tumor biological markers in postmenopausal breast cancer. Clin Cancer Res.

